# The Combined Administration of Vitamin C and Copper Induces a Systemic Oxidative Stress and Kidney Injury

**DOI:** 10.3390/biom13010143

**Published:** 2023-01-10

**Authors:** Rui Jiang, Yang Sui, Jingru Hong, Manabu Niimi, Qiaojing Yan, Zhuheng Shi, Jian Yao

**Affiliations:** 1 Division of Molecular Signaling, Department of the Advanced Biomedical Research, Interdisciplinary Graduate School of Medicine, University of Yamanashi, Chuo 409-3898, Japan; 2 Division of Molecular Pathology, Interdisciplinary Graduate School of Medicine, University of Yamanashi, Chuo 409-3898, Japan

**Keywords:** ascorbic acid, copper, oxidative stress, kidney injury, H_2_O_2_, antioxidant

## Abstract

Vitamin C (ascorbic acid; AA) and copper (Cu^2+^) are well used supplements with many health-promoting actions. However, when they are used in combination, the Fenton reaction occurs, leading to the formation of highly reactive hydroxyl radicals. Given that kidney is vulnerable to many toxicants including free radicals, we speculated that the in vivo administration of AA plus Cu^2+^ may cause oxidative kidney injury. The purpose of this study was to address this possibility. Mice were administered with AA and Cu^2+^, alone or in combination, via oral gavage once a day for various periods. Changes in the systemic oxidative status, as well renal structure and functions, were examined. The administration of AA plus Cu^2+^ elevated protein oxidation in serum, intestine, bladder, and kidney, as evidenced by the increased sulfenic acid formation and decreased level of free sulfhydryl groups (-SH). The systemic oxidative stress induced by AA plus Cu^2+^ was associated with a significant loss of renal function and structure, as indicated by the increased blood urea nitrogen (BUN), creatinine and urinary proteins, as well as glomerular and tubular cell injury. These effects of AA and Cu^2+^ were only observed when used in combination, and could be entirely prevented by thiol antioxidant NAC. Further analysis using cultured renal tubular epithelial cells revealed that AA plus Cu^2+^ caused cellular protein oxidation and cell death, which could be abolished by NAC and catalase. Moreover, coincubation of AA and Cu^2+^ led to H_2_O_2_ production. Collectively, our study revealed that a combined administration of AA and Cu^2+^ resulted in systemic oxidative stress and renal cell injury. As health-promoting supplements, AA and Cu^2+^ should not be used together.

## 1. Introduction

Vitamin C (Vit C), also known as ascorbic acid (AA), is an essential nutrient that cannot be produced by our body. Instead, the source of AA is foods and supplements. AA has many biological actions that make it indispensable for normal body function. It acts as a cofactor for several enzymes, regulating a wide range of cellular processes [1,2,3]. It is involved in the biosynthesis of L-carnitine, collagen, and neurotransmitters. In addition, it promotes the absorption of non-heme iron from the intestine and modulates iron transport and storage.

One of the critical biological functions of AA is its antioxidative action [1]. As an electron donor, AA is a redox-active chemical and readily participates in the redox reaction. It contributes to body defense against oxidative damage initiated by multiple stimuli. It has been reported that AA prevents the oxidation of low-density lipoproteins [4] and alleviates oxidative stress in the digestive and cardiovascular systems [5]. It has been widely used to treat various illnesses, such as cardiovascular diseases, the common cold, infections, and cancer [2]. The use of high doses of AA in clinical trials as a therapeutic agent to treat coronavirus infection and cardiovascular diseases is gaining attention [6,7]. Paradoxically, AA also has a prooxidative activity [1], especially when used at high concentrations. AA has been shown to undergo autoxidation in the presence of transition metal ions (copper and iron), forming ascorbate free radical and dehydroascorbic acid via two successive one-electron oxidations, along with the generation of ROS (O_2_•, H_2_O_2_, and OH•) [8,9]. This property of AA has been used in cancer therapy [10]. The infusion of megadoses of AA (up to 4 g/kg) has been used in tumor therapy in animal models and patients [11,12]. Given that oxidative stress (OS) is one of the most common mechanisms underlying various diseases [13], the prooxidant property of AA could have serious consequences. However, although there are several papers documenting a cytotoxic action of AA at pharmacological concentrations on cultured cells, the in vivo toxicity of vitamin C has not been thoroughly tested.

The kidney is the organ responsible for filtration, secretion, reabsorption, and ultimately excretion of various chemicals, including nephrotoxicants. It is critically involved in maintaining body homeostasis [14]. At the same time, it is also vulnerable to the toxicity of chemicals, especially heavy metals, because of its ability to reabsorb, accumulate and concentrate divalent metals [15].

Copper (Cu^2+^) is also a health supplement. It participates in many cellular processes [16]. A concentration of Cu^2+^ is nephrotoxic [17,18,19]. It has been reported that the administered Cu^2+^ is highly concentrated in the kidney through Cu^2+^ transporters in renal tubular cells [20]. Renal diseases are associated with an elevated Cu^2+^ transporter 1 (CTR1), the major transporter for Cu^2+^ influx [17]. The Cu^2+^ concentration in the kidney correlates with the severity of renal sclerosis, and chelation of Cu^2+^ is effective in inhibiting renal fibrosis [17,19,21]. Based on these considerations, we speculated that AA and Cu^2+^ could lead to oxidative renal injury. This study was designed to address this hypothesis.

We present here our data showing that AA and Cu^2+^ in combination caused systemic oxidative stress and oxidative renal cell injury. Our study thus indicates that the simultaneous administration of AA and Cu^2+^ should be avoided.

## 2. Materials and Methods

### 2.1. Materials

Copper(II) Sulfate (CuSO_4_; Cu^2+^) was purchased from FUJIFILM Wako Pure Chemical Corporation (Osaka, Japan). Bovine serum albumin (BSA, Faction V) was obtained from Iwai Chemical Company (Tokyo, Japan). Dimedone was purchased from Tokyo Chemical Industry (Tokyo, Japan). Anti-cysteine sulfenic acid antibodies were acquired from Millipore (Burlington, MA, USA). HRP-conjugated anti-rabbit was purchased from Cell Signaling, Inc. (Beverly, MA, USA). The Alexa 680 Fluor C2 maleimide and blood urea nitrogen (BUN) kit were from Thermo Fisher Scientific (Rockford, IL, USA). L-Ascorbic acid (AA), maleimide, N-Acetyl-L-cysteine (NAC) and catalase polyethylene glycol and all other chemicals were acquired from Sigma-Aldrich (St. Louis, MO, USA).

### 2.2. Cell Culture

Rat renal tubular epithelial NRK-52E cells were purchased from the American Type Culture Collection (ATCC, Rockville, MD, USA). Cells were cultured in DMEM/F12 (Gibco-BRL, Gaithersburg, MD, USA) containing 5% FBS and 1% antibiotic and antimycotic solution in a humidified atmosphere of 5% CO_2_/95% air at 37 °C. For experiments, cells were seeded into the 12- or 96-well culture plate in DMEM/F12 containing 1% FBS and exposed to various stimuli.

### 2.3. Animal Experiments

Both male and female 14–19-week-old C57BL6/J mice were used. Mice were housed in quiet rooms with a 12-h light-dark cycle and allowed to access food and water freely. For experiments, mice were divided into either four groups (control group, AA group, Cu^2+^ group, or Cu^2+^ plus AA groups), or two groups (control group or AA plus Cu^2+^ group), each with 4–5 mice. AA at the doses ranging from 10 mg/kg to 1000 mg/kg and Cu^2+^ at 1 mg/kg was administered via oral gavage for the indicated period. The same volume of saline was used as the untreated control. Of note, in most animal experiments, AA and Cu^2+^ have been administered at 100 mg/kg and 1 mg/kg, respectively. The doses were relatively low as compared to the previous reports [11,12,22,23] to avoid the potential unfavorable effects arising from either side of AA or Cu^2+^. Urine, feces and blood were collected and stored at −80 °C. At the end of the experiments, mice were anesthetized by the intraperitoneal injection of sodium pentobarbital (40 mg/kg) (somnopentyl^®^, Kyoritsu seiyaku corp., Tokyo, Japan), and organs (kidney, bladder and colon) were taken and stored at −80 °C until further analysis. The animal study was approved by the Animal Care and Use Committee of Yamanashi University (A3-583) and carried out according to the guideline and rules for animal experimentation.

### 2.4. Western Blot Analysis

Cellular proteins were extracted using a 1 × SDS lysis buffer (62.5 mM Tris-HCl, 2% SDS, 10% glycerol), and tissue samples were homogenized in a RIPA lysis buffer together with a freshly added proteinase inhibitor cocktail. After protein determination with the Micro BCA Protein Assay Kit (Thermo Fisher Scientific, Waltham, MA, USA), an equal amount of proteins was loaded into 10% SDS-PAGE, separated and transferred to PVDF membranes with a wet-blotting apparatus. After blocking with 5% skimmed milk or 3% BSA in 0.1% Tween-20 PBS solution (TPBS) for 1 h, the membrane was incubated overnight with the first antibody (nephrin, Cat. #sc-28192; podocin, Cat. #sc-21009; Santa Cruz Biotechnology) at the appropriate dilution. After washing with TPBS, the blots were incubated with a peroxidase-conjugated secondary antibody for 1 h and detected for the target proteins using the enhanced chemiluminescence system (Nacalai Tesque, Kyoto, Japan). The chemiluminescent signal was captured with a Fujifilm image LAS-1000 analyzer (Fujifilm, Tokyo, Japan). The intensity of the bands was quantified with NIH Image J software (http://rsb.info.nih.gov/ij, accessed on 11 December 2022). To confirm the equal loading of samples in each lain, EZ blue staining of the membrane protein or probing the blot with β-actin was performed.

### 2.5. Detection of Sulfenic Acid

The protein level of sulfenic acid formation was detected using the method as previously reported [24,25]. Briefly, proteins extracted from cultured cells, bladder, colon and kidney at the concentration of 1–3 mg/mL were treated with 1 mM dimedone for 20 min at RT. After mixing with 1/5 volume of 5X non-reducing sample buffer, samples were subjected to Western blot analysis for sulfenic acid with an anti-dimedone antibody (Millipore; Cat. #07-2139).

### 2.6. Cellular Viability Assay

Cells grown in 96-well culture plates were exposed to water-soluble tetrazolium salt, WST-8, and reagent (Dojindo, Kumamoto, Japan) for 60 min. The optical density (OD) at the wavelength of 450 nm was measured with a spectrometer (SpectraMax 340, Sunnyvale, CA, USA).

### 2.7. Lactate Dehydrogenase (LDH) Release

LDH release was measured with a commercial kit following the manufacturer’s instructions (LDH Cytotoxicity Detection Kit, TaKaRa). Briefly, cells were seeded into a 96-well plate and grown to 80–90% confluence in DMEM/F12 containing 1% FBS. After exposure to various stimuli for the indicated time intervals, the supernatant was collected and allowed to incubate with the same volume of assay buffer at RT for 30 min. The intensity of the developed red color was measured with a spectrometer at 490 nm. LDH release was expressed as the percentage of the total release achieved by incubating cells with 2% Triton X-100 for 10 min.

### 2.8. Calcein-AM/Propidium Iodide (PI) Staining 

The Calcein-AM/PI live/dead staining was performed using an assay kit from (Dojindo, Kumamoto, Japan). Briefly, cells were incubated with a mixture of Calcein-AM (green) and PI (red) solution for 10–20 min. The living Calcein-AM positive green cells and dead PI-positive red cells were photographed under a fluorescence microscope.

### 2.9. Histochemical Staining of Kidney 

A kidney histology was conducted, as previously reported [25]. Briefly, kidney tissues were fixed with 10% formalin, embedded in paraffin, sliced into 4-μm sections, and stained following the standard hematoxylin and eosin (H&E) procedure. The section was observed and photographed with an Olympus Inverted Microscope (IX71, Olympus, Tokyo, Japan) at 200× magnification.

### 2.10. Determination of H_2_O_2_ Concentration

The level of H_2_O_2_ was detected using a kit from Cayman Chemical Company (MI, USA, 600050) following the protocol provided by the manufacturer. Briefly, samples (PBS control, AA and Cu^2+^ at the indicated concentrations) were added into a 96-well plate and allowed to react with an assay buffer and enzyme reaction solution for 30 min. The developed fluorescence was measured with a SpectraMax^®^ GEMINI EM (Sunnyvale, CA, USA) at the excitation wavelength of 530 nm and an emission wavelength of 590 nm.

### 2.11. Maleimide-Labelling Assay 

A maleimide-labeling assay for -SH groups was performed as previously reported [25,26,27]. Proteins from feces, serum and organs (kidney, bladder and colon) were allowed to react with 5 μM Alexa Fluor 680 C2 maleimide at 4 °C for 2 h. After the reaction, the protein samples were either subjected to SDS-PAGE separation or directly applied to the PVDF membrane in a Bio-Rad dot-blot apparatus. The fluorescent signals of the labeled maleimide in the gel or membranes were captured with a Fujifilm image LAS-4000 analyzer (Fujifilm, Tokyo, Japan) and quantified with ImageJ software. The equal loading of proteins in each lane was verified by EZ blue staining.

### 2.12. Measurement of BUN, Creatinine and Urinary Proteins 

Blood urea nitrogen (BUN) and creatinine were measured with commercial kits following the protocols provided by the manufacturers. Briefly, sera with or without dilution were allowed to react with the assay buffer for the required period. The optical absorption was measured at 450 nm for BUN and 490 nm for creatinine with a spectrometer (SpectraMax 340). Urinary proteins were measured using a Micro BCA assay kit following the manufacturer’s instructions (Thermo Fisher Scientific, Rockford, IL, USA).

### 2.13. Statistic 

Values are expressed as mean ± SE. A comparison of two groups was made by Student’s *t*-test. For multiple comparisons with the same control, one-way ANOVA analysis and post hoc comparisons (Dunnett’s test and Tukey’s test) were performed. Both analyses were done using Microsoft Excel (Microsoft, Redmond, WA, USA) or GraphPad Prism 9. *p* < 0.05 was considered statistically significant.

## 3. Results

### 3.1. Combined Administration of Ascorbic Acid (AA) and Copper (Cu^2+^) Induces Systemic Oxidative Stress

**Figure 1:**
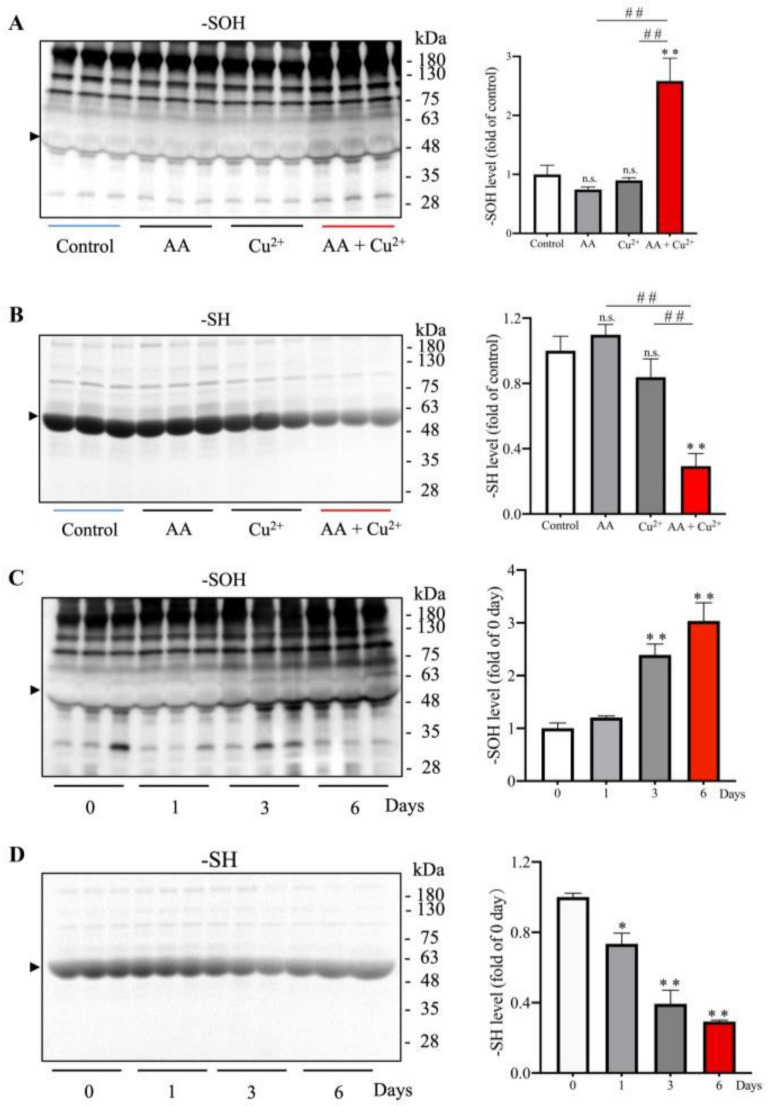
Effects of the in vivo administration of ascorbic acid (AA) and copper (Cu^2+^) on the oxidative status of -SH groups in serum proteins. (**A**,**B**) Mice were administered 100 mg/kg AA or 1 mg/kg Cu^2+^ or both in combination via oral gavage once a day for five consecutive days. (**C**,**D**) Mice were administered with AA plus Cu^2+^ for the indicated time. Serum proteins were analyzed for the level of sulfhydryl groups (-SH) and sulfenic acids (-SOH) formation. The densitometric quantitation of the bands around 50~60 kDa (arrowhead) were performed and presented as the bar graph on the right side of the blot. Data shown are mean ± SE (*n* = 3–4; n.s.: not significant; * *p* < 0.05; ** *p* < 0.01 vs. control; ^##^ *p* < 0.01).

Given that AA and Cu^2+^ in combination lead to the generation of H_2_O_2_ and a hydroxyl radical (OH•) via Fenton reaction [8,9], we tested whether in vivo administration of AA and Cu^2+^ could lead to systemic oxidative stress. For this purpose, we have administered the mice with 100 mg/kg AA and 1 mg/kg Cu^2+^, alone or in combination, through gavage once per day for 5 days. The levels of oxidative status in proteins from blood, the intestines, and bladder were examined. Figure 1 shows that the in vivo administration of AA plus Cu^2+^ elevated the level of sulfenic acids (-SOH) and decreased free sulfhydryl groups (-SH) in several serum proteins. This effect was especially pronounced at the location around 50~60 kDa, corresponding to the predicted MW of serum albumin (arrowhead), which is known to be the predominant source of -SH groups in serum [25,28]. Fascinatingly, this effect of AA and Cu^2+^ was only observed in mice receiving both AA and Cu^2+^, but not AA or Cu^2+^ alone (Figure 1A,B). A time-course analysis revealed that the apparent changes could be observed on day 3 and sustained for the whole observation period (Figure 1C,D).

**Figure 2:**
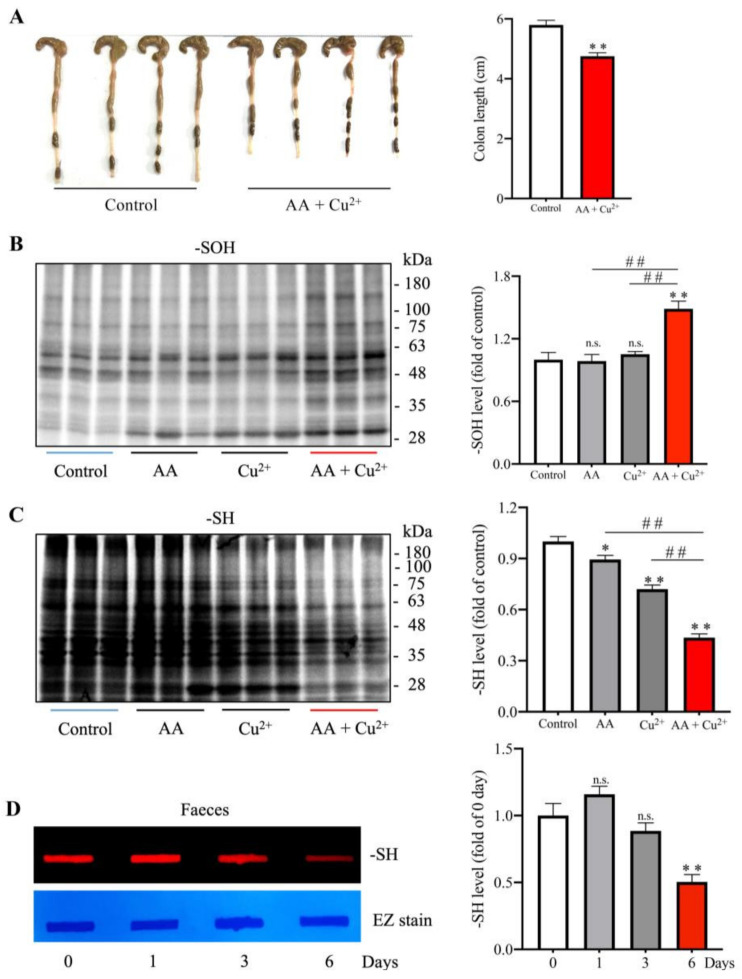
Effects of in vivo administration of AA and Cu^2+^ on colon length and oxidation. (**A**) Effects of combined administration on colon length. Mice were administered 100 mg/kg AA or 1 mg/kg Cu^2+^ in combination via oral gavage once a day for 6 days. The colon was taken, photographed (**A**), and measured for length. The results shown on the right side of the photo are mean ± SE (*n* = 4–6; ** *p* < 0.01 vs. control). (**B**,**C**) Effects of AA plus Cu^2+^ on the oxidative modification of -SH groups in colon proteins. Mice were administered with 100 mg/kg AA or 1 mg/kg Cu^2+^ or both in combination via oral gavage once a day for five consecutive days. The level of -SH groups and -SOH formation in colon lysates were detected. The quantitation of the bands was performed, and data are shown in the bar graph below the blot. Data shown are mean ± SE (*n* = 3–4; n.s.: not significant; * *p* < 0.05; ** *p* < 0.01 vs. control; ^##^ *p* < 0.01). (**D**) Effects of AA plus Cu^2+^ administration on the level of -SH groups in feces. Proteins from feces were subjected to Dot blot analysis for the level of -SH groups using a maleimide-labeling assay. The result of the densitometric analysis of the bands is shown at the right side of the blot. Data shown are mean ± SE (*n* = 3, n.s.; ** *p* < 0.01 vs. control).

Because the digestive system is responsible for the absorption of AA and Cu^2+^, it should be the major organ to be affected. Figure 2A shows that colon length in AA and Cu^2+^ administered mice was significantly shortened compared to the control. Western blot analysis of colon lysates revealed that the level of oxidized proteins was increased, whereas the level of -SH groups was reduced (Figure 2B,C). Consistently, the -SH level in feces was also significantly reduced, as revealed by the reduced fluorescence of the maleimide-labeled band in dot-blot (Figure 2D).

As an organ that stores urine for a certain period until urination, the bladder could also be affected. Indeed, AA and Cu^2+^ in combination also altered the oxidative status in the bladder (Supplementary Figure S1). These observations indicate that the combined administration of AA and Cu^2+^ induces systemic oxidative stress.

### 3.2. AA and Cu^2+^ in Combination Cause Renal Injury 

**Figure 3:**
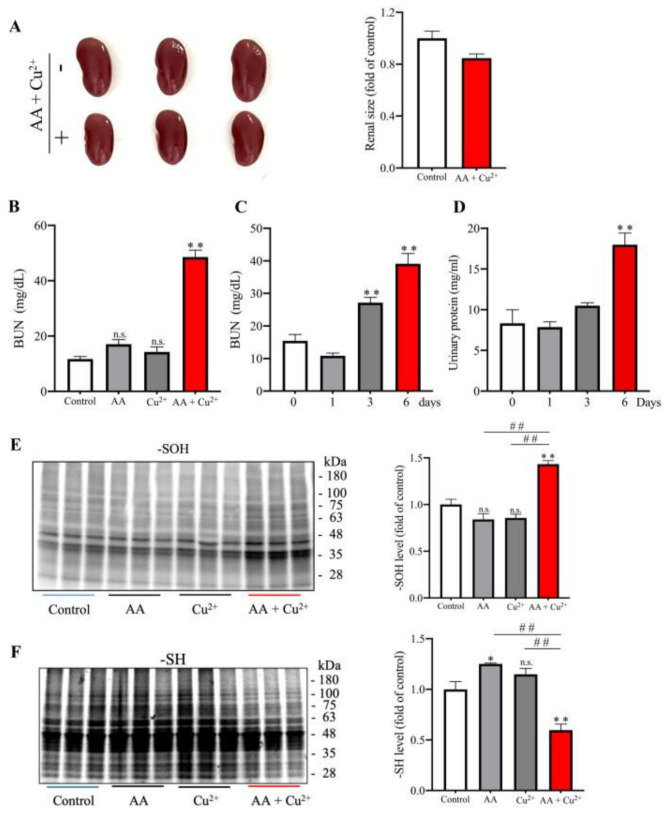
Effects of in vivo administration of AA and Cu^2+^ on the kidney. (**A**) Effects of AA plus Cu^2+^ on kidney size. Mice were administered 100 mg/kg AA or 1 mg/kg Cu^2+^ or both in combination via oral gavage once a day for six days. The kidneys were photographed and measured for their sizes (area). The data shown on the right side are % changes of renal size relative to control (mean ± SE; *n* = 3). (**B**–**D**) Effects of AA plus Cu^2+^ on the level of BUN and urinary proteins. Mice were administered 100 mg/kg AA or 1 mg/kg Cu^2+^ or both in combination via oral gavage once a day for five (**B**) or six days (**C**,**D**). The level of BUN and urinary proteins was measured. Data shown are mean ± SE (*n* = 3–6; n.s.: not significant; ** *p* < 0.01 vs. control). (**E**,**F**) Effect of AA and Cu^2+^ on the level of -SOH formation and -SH groups in the kidney. The result of the densitometric analysis is shown on the right side of the blot. Mice were administered with 100 mg/kg AA or 1 mg/kg Cu^2+^ or both in combination via oral gavage once a day for five days. The level of -SOH formation and -SH groups in kidney lysates were measured. Data shown are mean ± SE (*n* = 3–4; n.s.: not significant; * *p* < 0.05; ** *p* < 0.01 vs. control; ^##^ *p* < 0.01).

Given that kidney is an organ vulnerable to various toxicants because of its role in infiltration, absorption and excretion [14], we asked whether AA and Cu^2+^ could lead to renal injury. For this purpose, we evaluated renal changes in size, function, structure, and oxidative status. Figure 3A shows that kidney size tended to be decreased in mice receiving AA plus Cu^2+^ for six days. A biochemical analysis revealed that AA plus Cu^2+^ elevated BUN and urinary protein, indicative of a loss of renal function (Figure 3B–D). Intriguingly, this effect was only observed in AA plus Cu^2+^, but not AA or Cu^2+^ alone (Figure 3B). Consistently, only Cu^2+^ and AA in combination caused a significant change in the oxidative status of -SH groups in kidney (Figure 3E,F).

**Figure 4:**
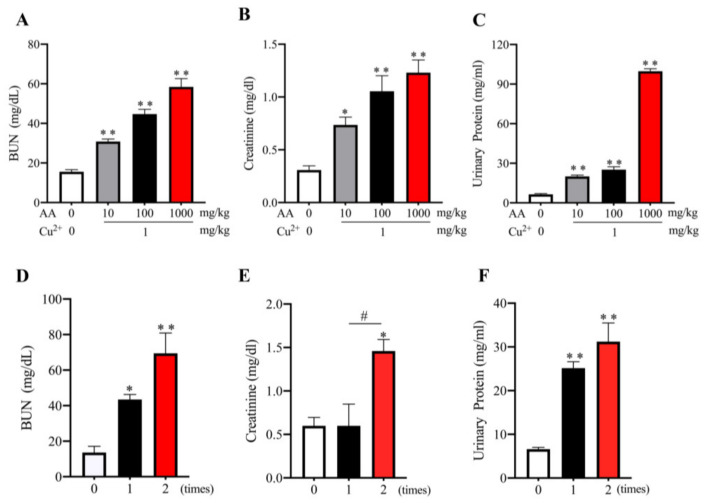
Dose- and time-effects of AA and Cu^2+^ on kidney functions. Mice were administered with the indicated concentrations of AA together with or without 1 mg/kg Cu^2+^ via oral gavage once a day (**A**–**C**) or with 100 mg/kg AA plus 1 mg/kg Cu^2+^ once or twice per day for six days (**D**–**F**). The level of BUN, creatinine and urinary proteins were measured. Data shown are mean ± SE (*n* = 4; * *p* < 0.05; ** *p* < 0.01 vs. control; ^#^ *p* < 0.05).

Further analysis revealed that the effect of AA plus Cu^2+^ on the kidney was closely related to AA concentrations (Figure 4A–C). AA at a concentration as low as 10 mg/kg was enough to induce a significant loss of renal function. In addition, the administration of AA plus Cu^2+^ twice per day resulted in a more severe loss of renal function than once per day (Figure 4D–F), suggesting that the effect of AA was time- and concentration-dependent.

**Figure 5:**
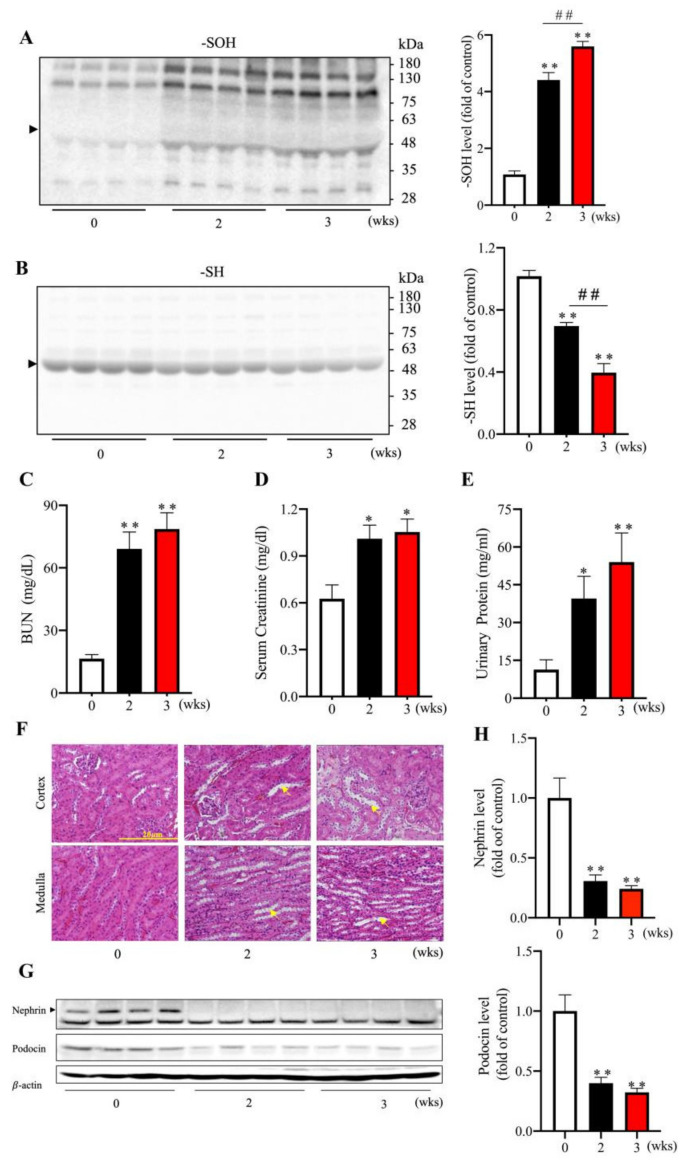
Long-term administration of AA and Cu^2+^ on renal structure and functions. (**A**,**B**) Effects of long-term administration on serum proteins oxidation. Mice were administered 100 mg/kg AA or 1 mg/kg Cu^2+^ or both in combination via oral gavage once a day for two and three weeks. Serum was collected and analyzed for the level of -SH groups and -SOH formation. The densitometric quantitation of the bands around 50~60 kDa (arrowhead) was performed and presented as the bar graph on the right side of the blot. Data shown are mean ± SE (*n* = 4; ** *p* < 0.01 vs. control; ^##^ *p* < 0.01). (**C**–**E**) Effects of AA plus Cu^2+^ on renal functions. The level of BUN, creatinine and urinary proteins were measured. The data shown are mean ± SE (*n* = 4–8; * *p* < 0.05; ** *p* < 0.01 vs. control). (**F**–**H**) The effect of AA plus Cu^2+^ on renal histopathological changes and podocyte injury markers. Representative images of HE staining of the kidney are shown in (**F**). Note that AA plus Cu^2+^ destroyed the intact epithelial architecture, including the loss of brush border, detachment of epithelial cells, and tubular cell vacuolation (arrow). The effect of AA plus Cu^2+^ on the podocyte injury markers nephrin and podocin is shown in (**G**,**H**). Data shown in (**H**) are mean ± SE (*n* = 4; ** *p* < 0.01 vs. control).

We also observed the long-term effect of AA plus Cu^2+^ on oxidative status and renal functions. The administration of AA plus Cu^2+^ for two or three weeks caused a time-dependent elevation in serum protein oxidation (Figure 5A,B) and loss of renal functions (Figure 5C–E). A pathological analysis revealed that AA plus Cu^2+^ caused renal tubular damage, as evidenced by the appearance of tubular dilation, tubular cell vacuolation and detachment, especially at the outer medulla (Figure 5F). Other than the damages in tubular cells, AA plus Cu^2+^ also caused glomerular injury, as revealed by the significant reduction of the slit diaphragm proteins nephrin and podocin in Western blot analysis (Figure 5G,H). All of these observations suggest that the combined use of AA plus Cu^2+^ induces renal injury.

### 3.3. AA and Cu^2+^ in Combination Cause Cytotoxicity in Cultured Renal Cells 

**Figure 6:**
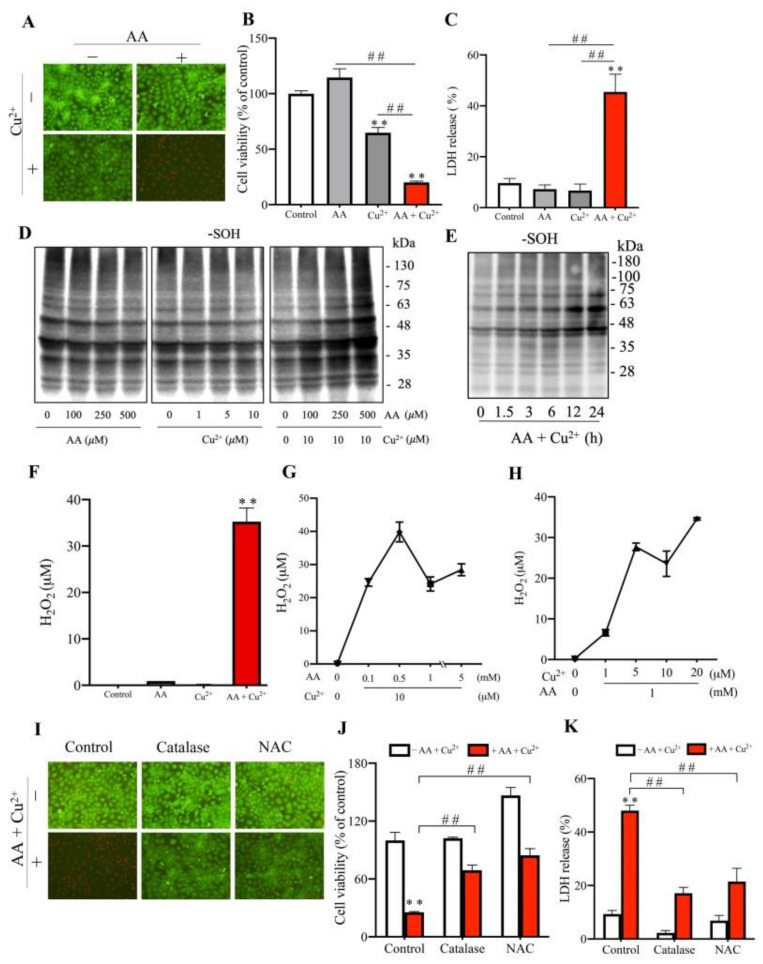
Effects of AA plus Cu^2+^ on renal tubular viability and cellular oxidative status. (**A**–**C**) Effects of AA and Cu^2+^ on renal tubular cell death. Renal tubular epithelial NRK cells were exposed to 1 mM AA, 10 μM Cu^2+,^ or AA plus Cu^2+^ in combination. The cell viability was evaluated by Red/Green staining (**A**), formazan formation (**B**) and LDH release (**C**). The data shown in (**B**) and (**C**) are expressed as relative changes against control in (**A**) and % of total LDH release in (**B**). Data shown are mean ± SE (*n* = 4; ** *p* < 0.01; ^##^ *p* < 0.01). (**D**,**E**) Effect of AA and Cu^2+^ on protein oxidation. Cells were treated with the indicated concentrations of AA or Cu^2+^ for 24 h (**D**), or 500 μM AA and 10 μM Cu^2+^ for the indicated time (**E**). Cellular lysates were subjected to Western blot analysis for sulfenic acid formation. (**F**–**H**) Effect of AA and Cu^2+^ coincubation on H_2_O_2_ generation. One mM AA and 10 μM Cu^2+^ or the indicated concentrations of AA and Cu^2+^ (**G**,**H**) were allowed to react for 30 min. The level of H_2_O_2_ was measured using a commercial kit as described in the material and methods section. Data shown are mean ± SE (*n* = 4; ** *p* < 0.01 vs. control). (**I**–**K**) The prevention of AA and Cu^2+^-induced renal tubular injury by antioxidants. Cells were exposed to AA plus Cu^2+^ for 12 h in the presence or absence of 100 unit/mL catalase or 2 mM NAC. The cell viability was determined by red/green staining (**I**), formazan formation (**J**), and LDH release (**K**). The results shown in (**J**,**K**) are the percentage of control and percentage of 100% LDH release, respectively. Data shown are mean ± SE (*n* = 4, ** *p* < 0.01; ^##^ *p* < 0.01).

To further confirm the effect of AA plus Cu^2+^ and to explore the underlying mechanisms, we have examined the effect of AA and Cu^2+^ on cultured renal tubular epithelial cells. Figure 6A–C shows that the exposure of renal tubular epithelial NRK cells to AA plus Cu^2+^ resulted in cell injury, as evidenced by the appearance of PI-positive red dead cells, the reduced amount of formazan formation, as well as the increased LDH release. This effect of AA plus Cu^2+^ was associated with a marked increase in cellular protein oxidation (Figure 6D,E). 

Further analysis revealed that the direct incubation of AA with Cu^2+^ caused H_2_O_2_ generation. The amount of H_2_O_2_ produced appeared to be dependent upon both AA and Cu-concentration (Figure 6F–H). In support of a mediating role of the generated H_2_O_2_ on AA plus Cu^2+^-induced renal cell injury, we have used the H_2_O_2_ scavengers catalase and NAC and found that these chemicals largely abolished the cytotoxic effect of AA plus Cu^2+^ (Figure 6I–K). Of note, consistent with in vivo observation, AA and Cu^2+^ alone did not affect cell survival, oxidation, or H_2_O_2_ generation. Collectively, these observations indicate that AA plus Cu^2+^ causes renal cell injury via mechanisms involving H_2_O_2_ formation and protein oxidation.

### 3.4. Antioxidant NAC Protects the Kidney from AA Plus Cu^2+^-Induced Renal Injury

**Figure 7:**
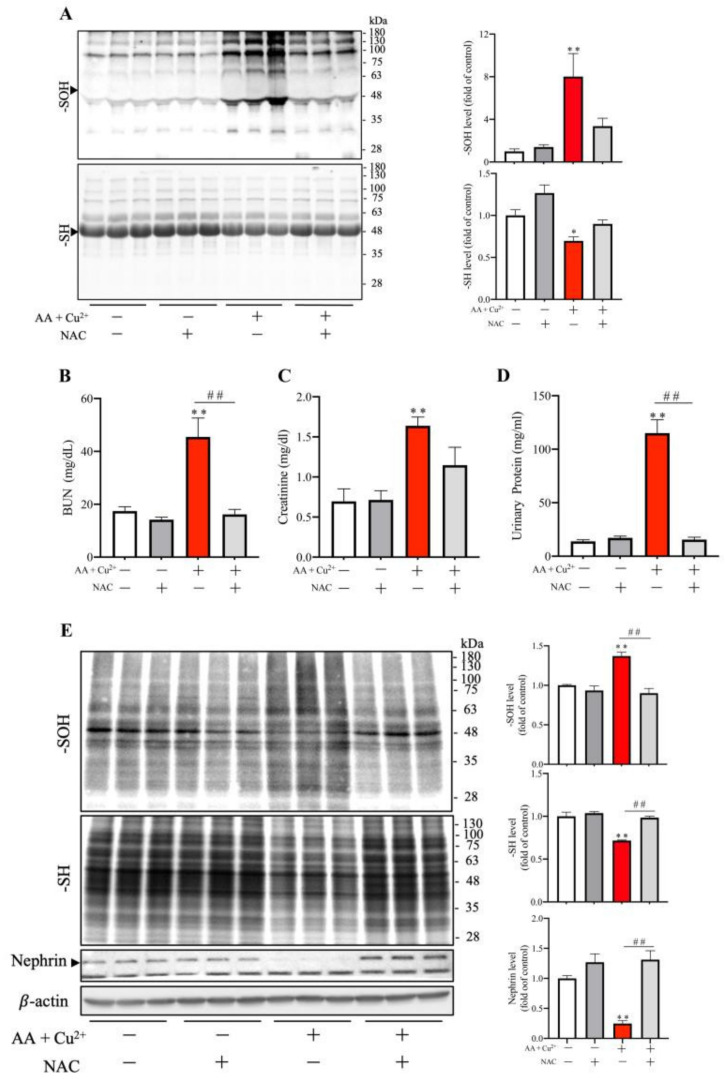
Prevention of AA and Cu^2+^-induced systemic oxidative stress and renal injury by antioxidant NAC. Mice were intraperitoneally injected with 200 mg/kg NAC at 12-h intervals for 6.5 days. AA (1000 mg/kg) plus Cu^2+^ (1 mg/kg) were administered 12 h after the initial NAC injection via gavage once per day for six days. Serum proteins were analyzed for the level of -SH groups and -SOH formation (**A**). The densitometric quantitation of the bands around 50~60 kDa (arrowhead) was performed and presented as the bar graph on the right side of the blot. Data shown are mean ± SE (*n* = 3; * *p* < 0.05; ** *p* < 0.01 vs. control). BUN, creatinine and urinary protein levels were measured (**B**–**D**). Data shown are mean ± SE (*n* = 4; ** *p* < 0.01 vs. control, ^##^ *p* < 0.01). The level of -SOH/-SH and nephrin in kidney lysates was detected (**E**). The densitometric analysis of the band in each lane is shown on the right side of the blot. Data shown are mean ± SE (*n* = 3, ** *p* < 0.01 vs. control; ^##^ *p* < 0.01).

To demonstrate the mediating role of oxidative stress in AA plus Cu^2+^-induced kidney injury in vivo, we have treated the mice with 200 mg/kg NAC twice per day before and after exposing them to 1000 mg/kg AA plus 1 mg/kg Cu^2+^ for six consecutive days. Figure 7A shows that NAC treatment significantly prevented serum -SOH formation. It consistently reversed the decreased level of -SH groups in serum proteins caused by AA plus Cu^2+^. In addition, NAC also entirely prevented the loss of renal function (Figure 7B–D), as well as renal podocyte damage induced by AA plus Cu^2+^ (Figure 7E). This effect of NAC was also associated with an improved renal redox status in the kidney. These observations thus indicate that the nephrotoxicity induced by AA plus Cu^2+^ is mediated by oxidative stress.

## 4. Discussion

**Figure 8:**
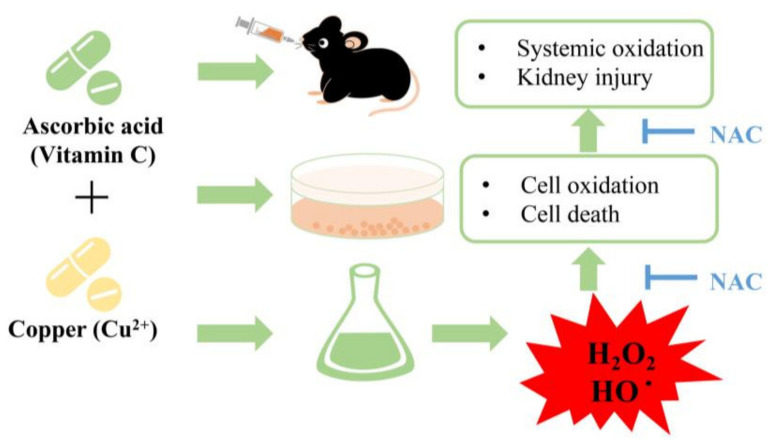
Schematic depiction of the effects and mechanisms of AA plus Cu^2+^ on systemic oxidative status and renal injury. The combined administration of AA plus Cu^2+^ resulted in H_2_O_2_ generation, which causes renal tubular cell injury in vitro and systemic oxidative stress as well as kidney injury in vivo. This effects of AA plus Cu^2+^ could be completely abolished by thiol antioxidant NAC.

In this study, we demonstrated, for the first time, that in vivo administration of AA plus Cu^2+^ caused systemic oxidative stress and kidney injury through mechanisms involving oxidative stress. The main findings have been schematically depicted in Figure 8. Our study provides direct evidence supporting the causative role of ROS in the initiation of renal diseases. It also serves as a warning for the potential health dangers of the combined use of AA and Cu^2+^. 

AA has also been shown to have prooxidant actions, especially at pharmacological concentrations [1,29]. This property of AA has been reported to be related to its bacterial- and tumor-killing actions [10,30,31,32]. Furthermore, it has been demonstrated that this action depends upon the presence of redox-active metal ions, such as iron and Cu^2+^ [10]. The underlying mechanism is thought to be due to the generation of H_2_O_2_ and the formation of the highly reactive hydroxyl radical. These radicals oxidize proteins, lipids, and nuclear acids, causing cell injury. In addition to its actions on cells, AA plus Cu^2+^ has also been reported to be able to oxidize biological proteins, such as immunoglobulin and albumin, in vitro [33,34]. Currently, most of the observations regarding the prooxidant actions of AA and Cu^2+^ have been performed in vitro. It is unclear whether it caused similar actions in vivo.

Here, we demonstrated that the in vivo administration of AA and Cu^2+^ caused systemic oxidative stress, as revealed by the elevated -SOH formation in serum protein and several different organs. Since sulfenic acid is the main product of the reaction between a protein thiol and hydrogen peroxide [24,25], the level of -SOH in serum and tissues should reflect the in vivo status of H_2_O_2_. In support of this conclusion, a previous study has shown that H_2_O_2_ could induce widespread -SOH formation in different tissues [24]. In this study, our in vitro results also support a critical involvement of H_2_O_2_ in AA plus Cu^2+^-induced oxidative stress and cell injury.

Besides oxidative effects on cells, AA plus Cu^2+^ oxidized biological proteins in vivo. Among oxidized serum proteins, one of the major proteins to be affected could be albumin. As the predominant protein in body fluid, albumin occupies more than 90% of the -SH groups and is vulnerable to the attack of H_2_O_2_ and hydroxyl radicals [28]. In agreement with these features, the protein to be dramatically oxidized was localized at the predicted MW of albumin and had the highest level of -SH groups. The finding about the in vivo oxidation of albumin by AA plus Cu^2+^ could have several implications. First, it has been well established that there exists a coordinated and integrated regulation of thiol activity between albumin and other thiol antioxidants via -SH groups/disulfide bond exchange. The redox status of thiol in albumin could be used to indicate the systemic oxidative status. Second, H_2_O_2_ is known to be able to diffuse in the extracellular space, transmitting oxidative cell injury. However, the distance of diffusion is limited [35,36,37]. The oxidized serum proteins could serve as an even more critical mediator transmitting the oxidative injury from the local source of H_2_O_2_ to the remote organs, such as kidney.

Kidney is the organ responsible for the filtration, reabsorption, concentration, and excretion of various chemicals, which makes it vulnerable to a wide range of nephrotoxic substances [38,39]. Furthermore, renal cells are also highly vulnerable to circulating ROS and oxidized proteins [40,41,42]. These features may explain why the kidney was seriously damaged by AA and Cu^2+^. The detailed mechanisms involved in renal injury could be multiple, including both direct and indirect actions. For the direct action, the absorption of the administered AA and Cu^2+^ by renal cells could lead to local ROS generation, causing oxidative stress and oxidative renal cell injury. In accordance with this view, previous studies have demonstrated the expression of several AA and Cu^2+^ transporters in renal tubular epithelial cells and have shown that the kidney is one of the major organs with concentrated AA and Cu^2+^ after their administration [3,21,43,44,45,46]. Indirectly, the oxidatively modified serum proteins caused by AA and Cu^2+^ could act as a mediator transmitting oxidative stress. Previous studies have established the oxidative albumin as a vital mediator contributing to the initiation and development of renal diseases [42,47,48,49].

Of note, other than the direct and indirect effects mentioned above, high AA concentrations have also been reported to induce cell injury via the depletion of intracellular GSH [50]. The scenario is that AA was rapidly converted to oxidized form (DHA) after administration, which could enter the cells through the GLUT-1 glucose transporter, subsequently consuming intracellular GSH and causing cell injury. Because the GLUT-1 transporter is also highly expressed in renal glomerular and tubular cells [51], this mechanism could also contribute to the observed renal cell injury.

AA has been used in a wide range of concentrations in the cells and animal experiments, as well as clinical trials [11,12]. Both pro- and antioxidative actions have been reported [1]. For example, AA at the concentration of 10~200 mg/kg protected the hepatotoxicity induced by several different insults through its antioxidative actions [52,53]. A higher concentration of AA has been shown to have a prooxidant action toward tumor cells [11,12]. Unexpectedly, we did not observe an obvious change in the amount of -SH groups in serum and cellular proteins, failing to support either a reductive or a prooxidative role of AA. Intriguingly, there was also a report showing that AA at the concentration of 250 mg/kg prevented hematobiochemical alterations, oxidative stress, and kidney damage induced by 300 mg/kg CuSO4 in broiler chickens [23], which is in complete opposition to our results. The reason for the discrepancy is unclear. It could be due to the different experimental settings, such as indicators used for monitoring the redox state, approaches for administrating drugs, as well as species of animals used in the investigation. Since AA alone did not cause any changes in the systemic oxidative state in the current investigation, it is suggested that the presence of Cu^2+^ could be a determining factor for the pro-oxidative actions of AA.

Of note, although coincubation of AA and Cu^2+^ caused a rapid elevation in H_2_O_2_ production, the changes in protein oxidation in vivo were detectable three days after consecutive administration. This reason for the discrepancy could be due to the presence of a strong antioxidative system in vivo, which counteracted the oxidative modification induced by ROS at the early stage of the administration. With prolonged administration, the balance shifted to the oxidative side; thus, protein oxidation occurred. Indeed, the effect of AA and Cu^2+^ on the systemic oxidative state is cumulative and progressive with increased time and number of administrations. In this context, the long-term administration of a small dose of AA and Cu^2+^ could still cause oxidative cell injury.

Our study has significant clinical and basic implications. First, our study demonstrated that the in vivo administration of AA and Cu^2+^ caused systemic oxidative stress and kidney injury through ROS-related mechanisms. This property of AA and Cu^2+^ could be used to develop novel models of ROS-driven kidney injury, in which the role of ROS in the initiation and development of renal diseases could be clearly established. It also helps find effective anti-ROS therapeutics. Second, our study indicates that AA and Cu^2+^ should not be used concomitantly. Vitamin C and Cu^2+^ are well used health supplements, which are supposed to have many health benefits and to be easily available in the market. Our study indicates that Vitamin C and Cu^2+^ should not be used together.

## 5. Conclusions

In conclusion, the current study reveals that the combined administration of AA and Cu^2+^ resulted in systemic oxidative stress and oxidative renal injury. This property of AA and Cu^2+^ could be exploited to establish a presently unreported ROS-driven model of kidney disease. In addition, the findings from this study provides a warning for the health danger of the concomitant use of AA and Cu^2+^.

## Data Availability

The data is contained within the article.

## Supplementary Materials

The following supporting information can be downloaded at: https://www.mdpi.com/article/10.3390/biom13010143/s1. Effects of in vivo administration of Cu^2+^ and AA on the oxidative state of bladder. Effects of AA plus Cu^2+^ on the oxidative state of -SH groups of bladder proteins. Mice were administered 100 mg/kg AA or 1 mg/kg Cu^2+^ or both in combination via oral gavage once a day for consecutive 5 days. The level of -SH groups and -SOH in bladder lysates was detected. Densitometric analysis of the bands was performed, and the results are shown as the bar graph on the right side of the blot. Data shown are mean ± SE (n = 3–4; n.s.: not significant; ** *p* < 0.01 vs. control; ^##^ *p* < 0.01).
