# Supplementary material for: The Combined Administration of Vitamin C and Copper Induces a Systemic Oxidative Stress and Kidney Injury
Source: Biomolecules. 2023 Jan 10;13(1):143. doi: 10.3390/biom13010143 (PMC9856059; doi:10.3390/biom13010143)
Supplement: Supplementary file 1 [file biomolecules-13-00143-s001.zip › biomolecules-2129160-supplementary.pdf]

Supplementary Figure S1

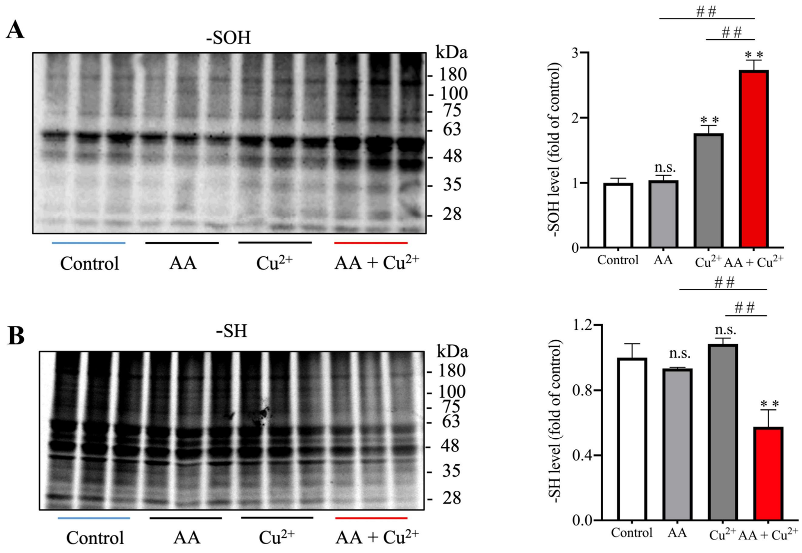

Supplementary Figure S1. Effects of *in vivo* administration of Cu^{2+} and AA on the oxidative state of bladder. Mice were administered 100 mg/kg AA or 1 mg/kg Cu^{2+} or both in combination via oral gavage once a day for consecutive 5 days. The level of -SOH (**A**) and -SH (**B**) in bladder lysates was detected. Densitometric analysis of the bands was performed, and the results are shown as the bar graph on the right side of the blot. Data shown are mean ± SE (n = 3-4; n.s.: not significant; ** $p < 0.01$ vs. control; $^{##}$ $p < 0.01$).
